# RUNX1T1 drives stem-like small-cell neuroendocrine prostate cancer identity

**DOI:** 10.3389/fcell.2026.1882758

**Published:** 2026-08-26

**Authors:** Ziqin Wang, Hyeryeon Jung, Changhyeon Hong, Nancy Chen, Yunsol Jo, Issac Jung, Taeeun Kim, Gina Lee, Keaton Chien, Ming Chen, Jiaoti Huang, Jung Wook Park

**Affiliations:** 1 Department of Pathology, Duke University School of Medicine, Durham, NC, United States; 2 Duke Cancer Institute, Duke University, Durham, NC, United States; 3 Department of Cell Biology, Duke University School of Medicine, Durham, NC, United States

**Keywords:** apoptosis, cell identity, human prostate cell reprogramming assay, neuroendocrine differentiation, RUNX1T1, stem-like small-cell neuroendocrine prostate cancer, stemness

## Abstract

Neuroendocrine prostate cancer (NEPC) or small-cell neuroendocrine prostate cancer (SCNPC) is increasingly recognized as a treatment-resistant disease arising among prostate adenocarcinoma (PRAD) patients. It has no effective therapy due to incomplete understanding of the mechanisms underlying treatment-induced cancer cell identity switching. Here, we identified RUNX1 Partner Transcriptional Co-Repressor 1 (RUNX1T1) as a frequently amplified gene in prostate cancer patients, associated with a poorer prognosis. RUNX1T1 is highly expressed and progression-correlated in NEPC and SCNPC. We found that RUNX1T1 is essential for SCNPC survival, as its knockdown induced apoptotic cell death and the acquisition of PRAD-like identity, including alterations in genes involved in cell-cell adhesion and epithelial cell polarity. Functional assays in prostate cancer cell lines revealed that RUNX1T1 regulates genes associated with stemness and the maintenance of neuroendocrine identity in SCNPC. Using our human cell reprogramming assay, which mimics the development of PRAD or SCNPC *in vivo*, we further demonstrated that RUNX1T1 is required to initiate neuroendocrine differentiation (NED), histological feature changes, and stemness. Its knockout abolished expression of NED and stemness markers and induced loss of SCNPC histological features during SCNPC development. Collectively, these findings establish RUNX1T1 as a critical driver of the cell identity of stem-like SCNPC. Understanding the mechanisms by which RUNX1T1 exerts its versatile regulation of proliferation, stemness, and SCNPC differentiation will in turn pave the way for the discovery of new, specific therapeutic targets to prevent SCNPC progression.

## Introduction

Prostate cancer, the fifth leading cause of death and the second most prevalent cancer in men, has become a great health concern worldwide (Rawla, 2019). Early-stage prostate adenocarcinoma (PRAD) can be treated by surgery, radiation, and hormonal therapy, given its androgen-dependent nature (Litwin and Tan, 2017; Rawla, 2019). However, neuroendocrine prostate cancer (NEPC), characterized by loss of androgen receptor (AR) signaling and gain of neuroendocrine differentiation (NED) markers (CHGA, SYP, and NCAM1) (Terry and Beltran, 2014) eventually emerges to bypass the hormonal therapy in 17%–30% of castration-resistant prostate cancer (CRPC) patients (Litwin and Tan, 2017; Qi et al., 2023). Of all NEPC cases, small-cell neuroendocrine prostate cancer (SCNPC) is the most representative and aggressive subtype, characterized by a high nuclear-to-cytoplasmic ratio, scant cytoplasm, dense nuclei, extensive nuclear molding, and frequent mitotic figures (Beltran and Demichelis, 2021; Butler and Huang, 2021). The lack of a clinically approved targeted therapy for SCNPC, combined with its poor prognosis, poses a major clinical challenge (Aggarwal et al., 2018; Butler and Huang, 2021; Yamada and Beltran, 2021; Wishahi, 2024).

Efforts have been made to investigate the mechanisms of NED during prostate cancer progression. Key transcription factors such as ASCL1, FOXA2, NEUROD1, and PROX1 were identified to individually or collectively promote NED (Cejas et al., 2021; Han et al., 2022; Nouruzi et al., 2024; Rodarte et al., 2024). Other epigenetic modifiers, including EZH2 and LSD1, have been shown to play critical roles in mediating this lineage shift (Dardenne et al., 2016; Kumaraswamy et al., 2023; Venkadakrishnan et al., 2024). Studies have focused on the regulation of molecular signatures involved in the AR pathway and the NED program, while small-cell histology is often overlooked or generalized as a secondary effect of NED (Beltran et al., 2011; Beltran and Demichelis, 2021; Serritella et al., 2024). However, the occurrence of other NEPC subtypes with distinct histologic features, such as neuroendocrine carcinoid, further distinguished NED and small-cell identities as intertwined but distinct entities (Butler and Huang, 2021). The poorly differentiated histology and small cell sizes were often associated with distinct stem-like signatures in SCNPC (Li et al., 2015; Smith et al., 2015; Malta et al., 2018; Smith et al., 2018). Understanding the mechanisms underlying the histologic transformation from PRAD to SCNPC and the associated shifts in stemness identity may provide further clues for targeted therapy against SCNPC.

Herein, we identified RUNX1 Partner Transcriptional Co-Repressor 1 (RUNX1T1) to be a clinically relevant, highly upregulated gene in SCNPC. RUNX1T1 belongs to the myeloid translocation gene family, which includes members that share conserved domains to bind transcription factors, histone deacetylases, and corepressor complexes (Hu et al., 2022). Specifically, RUNX1T1 regulates the epigenome by recruiting histone deacetylases and DNA methyltransferases, while directly interacting with transcription factors to manipulate gene expression (Hu et al., 2022). A recent study revealed that RUNX1T1 is highly expressed in small-cell lung cancer (He et al., 2021), which shares common small-cell histology and NED features with SCNPC (Bluemn et al., 2017; Puca et al., 2019). However, the functional roles of RUNX1T1 in the NED and histologic transformation into SCNPC remained poorly understood. Therefore, we sought to examine the role of RUNX1T1 in regulating NED, histologic transformation, and stemness identity of SCNPC.

## Results

### RUNX1T1 is highly expressed in NEPC/SCNPC and is associated with disease progression

The progression of prostate adenocarcinoma (PRAD) to neuroendocrine prostate cancer (NEPC), particularly small-cell neuroendocrine prostate cancer (SCNPC), severely limits therapeutic options for patients with castration-resistant prostate cancer (CRPC). Identifying molecular drivers of this lineage transition may reveal therapeutic targets to prevent or suppress NEPC/SCNPC progression. To identify candidate regulators, we performed a meta-analysis integrating three independent datasets: single-cell RNA-seq from patients undergoing hormonal therapy (Dong et al., 2020), bulk RNA-seq data comparing NEPC and PRAD in CRPC patients (Beltran et al., 2016), and bulk RNA-seq data from enzalutamide-resistant PRAD cell lines (Verma et al., 2020).

**FIGURE 1:**
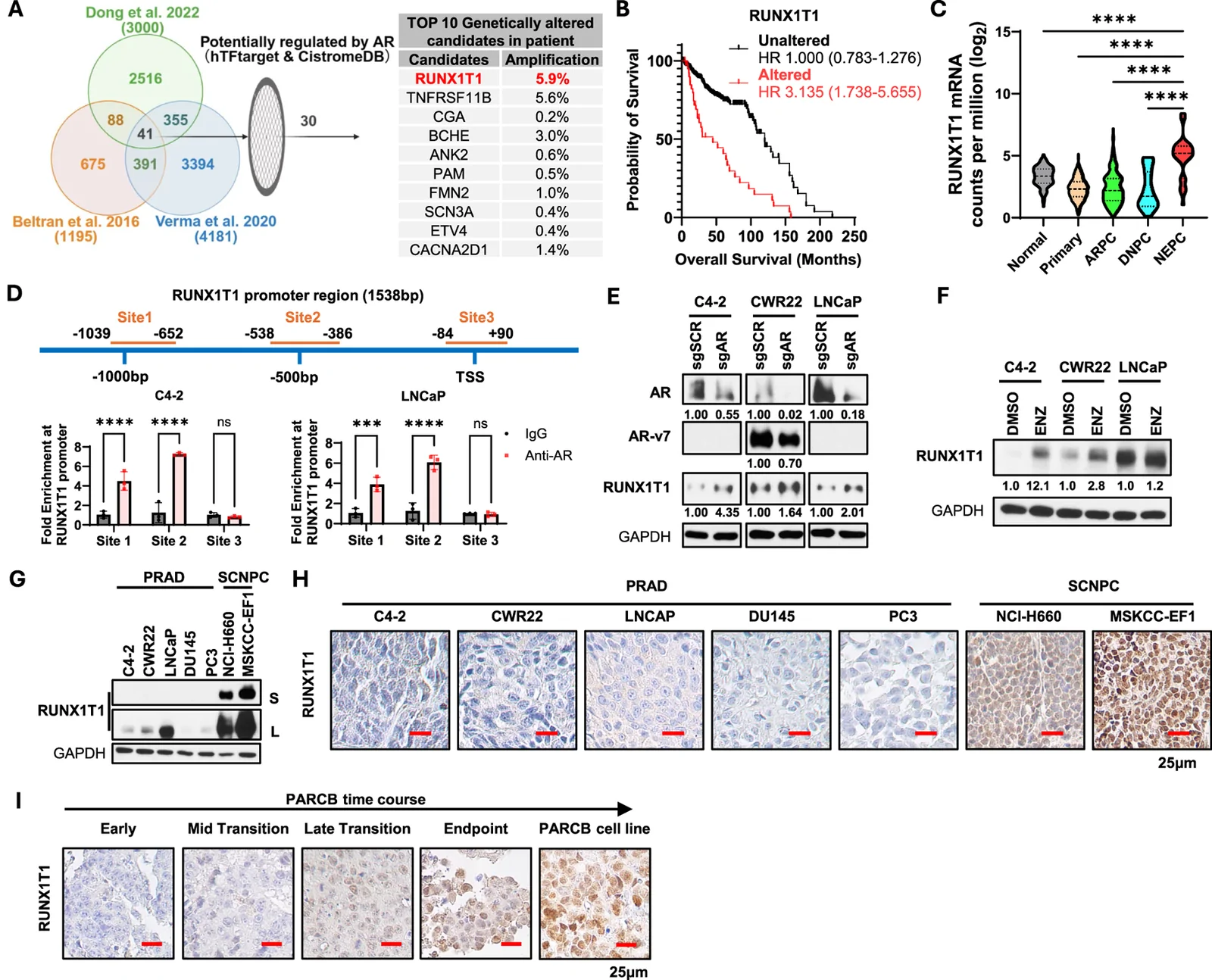
RUNX1T1 is highly expressed in NEPC/SCNPC and is associated with disease progression. **(A)** A schematic view of how RUNX1T1 was selected. By overlapping 3,000 dynamically expressed genes in NED (Dong et al., 2020), 1,195 genes upregulated in NEPC (FDR q < 0.001, fold change>3) (Beltran et al., 2016), and 4,181 genes upregulated in either ENZ-resistant C4-2B or LNCaP (FDR q < 0.05, fold change>5), 41 candidates were chosen. 30 of them were bound by AR in CistromeDB and hTF target database. RUNX1T1 is the top genetically altered or amplified gene in the combined analysis of all non-overlapped prostate cancer cohorts mapped to GRCh37/h19 in cBioPortal (Taylor et al., 2010; Barbieri et al., 2012; Cerami et al., 2012; Grasso et al., 2012; Baca et al., 2013; Gao et al., 2014; Hieronymus et al., 2014; Cancer Genome Atlas Research, 2015; Robinson et al., 2015; Beltran et al., 2016; Kumar et al., 2016; Abida et al., 2017; Fraser et al., 2017; Armenia et al., 2018; Gerhauser et al., 2018; Ren et al., 2018; Abida et al., 2019; Granlund et al., 2020; Nguyen et al., 2020; Stopsack et al., 2020; Chakraborty et al., 2022; Crowdis et al., 2022; Rodriguez-Calero et al., 2022; Stopsack et al., 2022; Tang et al., 2022; Anselmino et al., 2024; Lenis et al., 2024; Samueli et al., 2025). **(B)** Survival curve showing that RUNX1T1 alteration is associated with poor prognosis. Data accessed from cBioPortal (Cerami et al., 2012). Log-rank (Mantel-Cox) test, p < 0.0001. **(C)** Violin Plot showing that RUNX1T1 is upregulated along with prostate cancer progression from normal prostate to NEPC. Data accessed from Prostate Cancer Atlas www.prostatecanceratlas.com. Normal: normal prostate; Primary: primary prostate cancer; ARPC: castration resistant prostate cancer with AR expression; DNPC: prostate cancer double negative of AR and NE features; NEPC: neuroendocrine prostate cancer. Primary prostate cancer, ARPC, and DNPC are non-neuroendocrine prostate cancer. ANOVA, ****p < 0.0001. **(D)** Schematic view of primer sets targeting RUNX1T1 promoter and ChIP-qPCR result of AR against RUNX1T1 promoter in C4-2 and LNCaP. ANOVA, ***p < 0.001, ****p < 0.0001, ns: non-significant. TSS: transcription start site. **(E)** Immunoblot showing AR knockout increased RUNX1T1 expression in PRAD, along with quantification of fold changed relative to sgSCR controls. **(F)** Immunoblot showing Enzalutamide treatment for 14 days increased RUNX1T1 expression in PRAD, along with quantification of fold changed relative to DMSO controls. **(G)** Immunoblot showing RUNX1T1 is upregulated in SCNPC cell lines compared to PRAD cell lines *in vitro*. “S” represents shorter exposure; “L” represents longer exposure. **(H)** Representative images of IHC showing that RUNX1T1 is upregulated in SCNPC cell lines compared to PRAD cell lines *in vivo*. Scale bar: 25 μm. **(I)** Representative images of IHC showing that RUNX1T1 is upregulated gradually in human basal cell reprogramming model (PARCB) of SCNPC. Scale bar: 25 μm.

Because lineage plasticity and SCNPC transformation are frequently associated with suppression of androgen receptor (AR) signaling (Butler and Huang, 2021), we next interrogated CistromeDB and hTFtarget databases (Zheng et al., 2019; Zhang et al., 2020) to identify AR-associated genes with predicted AR binding at promoter or gene-body regions. Among the candidates, RUNX1T1 emerged as the most clinically relevant factor. RUNX1T1 exhibited recurrent genomic amplification in 5.9% of all prostate cancer patients (Figure 1A; Supplementary Figure 1A), with amplification frequencies reaching up to 28% in metastatic prostate cancer cohorts (Supplementary Figure 1B). Importantly, alterations in RUNX1T1 were associated with significantly worse overall survival (hazard ratio = 3.135, 95% CI: 1.738–5.655, p < 0.0001) (Figure 1B). Consistent with these findings, RUNX1T1 expression was low in normal prostate and non-neuroendocrine prostate cancer, but was significantly elevated in NEPC, supporting its association with disease progression and neuroendocrine differentiation (Figure 1C).

To determine whether RUNX1T1 is directly regulated by AR, as predicted by database analysis, we performed ChIP-qPCR in C4-2 and LNCaP cells. We observed greater than five-fold enrichment of AR occupancy at the RUNX1T1 promoter approximately 500 bp upstream of the transcription start site (TSS) (Figure 1D), supporting direct AR binding. Consistent with the established repressive role of AR signaling in lineage plasticity (Gritsina et al., 2019), disruption of AR signaling through either AR knockout or pharmacological inhibition with enzalutamide (ENZ) significantly increased RUNX1T1 expression in PRAD cell lines (Figures 1E,F). The high and specific expression of RUNX1T1 in AR-/NE+ compared to AR+/NE+ prostate cancer phenotypes (Labrecque et al., 2019), also corroborates its regulatory relationship with the androgen receptor (AR).

We further validated RUNX1T1 expression in SCNPC, the most aggressive and representative subtype of NEPC, across multiple models. RUNX1T1 protein levels were markedly elevated in SCNPC cell lines (NCI-H660 and MSKCC-EF1) compared with PRAD cell lines (C4-2, CWR22, LNCaP, DU145, and PC3) (Figures 1G,H). Moreover, our human prostate cell reprogramming model of SCNPC progression (Park et al., 2018; Chen et al., 2023) demonstrated a progressive increase in RUNX1T1 expression during the transition from early-stage lesions to advanced SCNPC (Figure 1I).

Collectively, these findings identify RUNX1T1 as a highly upregulated factor in NEPC/SCNPC that is associated with poor clinical outcome and disease progression, and suggest that RUNX1T1 may function as a key mediator of neuroendocrine lineage plasticity in prostate cancer.

### RUNX1T1 is required for SCNPC survival and promotes proliferation in PRAD

**FIGURE 2:**
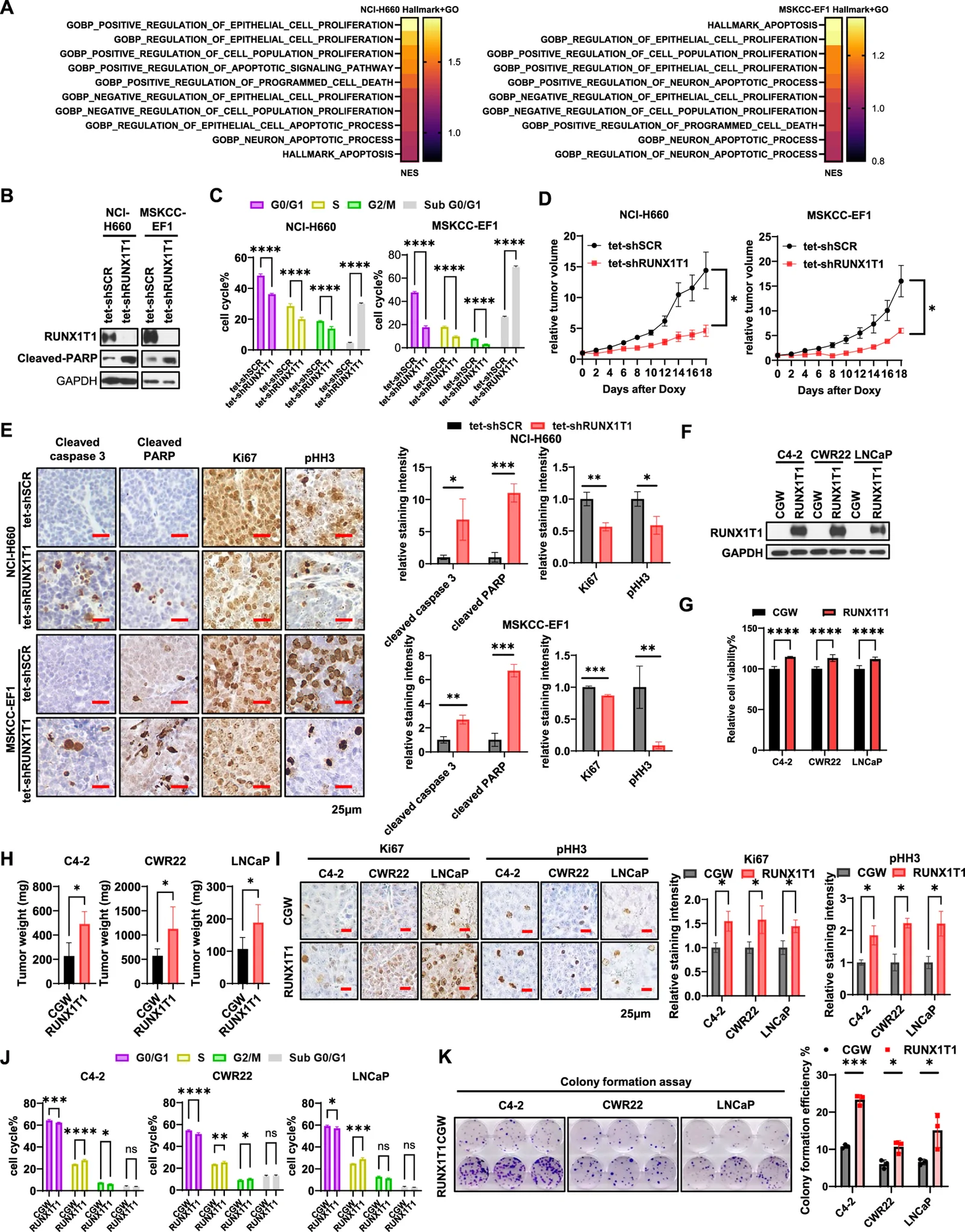
RUNX1T1 is required for SCNPC survival and promotes proliferation in PRAD. **(A)** Top 10 Hallmarks and GO terms associated with cell proliferation and apoptosis enriched in GSEA analysis of SCNPC tet-shRUNX1T1 DEGs compared to controls, suggesting an enrichment of apoptosis signature regulation of cell proliferation. NES values were generated by GSEA software. **(B)** Immunoblot of RUNX1T1 knockdown SCNPC compared to controls showing RUNX1T1 is efficiently knocked down and apoptosis marker (cleaved-PARP) is upregulated. **(C)** Cell cycle analysis in RUNX1T1 knockdown SCNPC compared to controls. ANOVA, 3 technical replicates, ****p < 0.0001. **(D)** Tumor growth curve showing tumor volume of RUNX1T1 knockdown SCNPC xenograft, normalized to the first day of Doxycycline induction. Student’s t-test of day 18 RUNX1T1 knockdown SCNPC compared to controls, 4 samples (tumors) per condition *p < 0.05. **(E)** Representative images of IHC of apoptosis markers (cleaved caspase3 and cleaved PARP) and proliferation markers (Ki67 and pHH3) in RUNX1T1 knockdown SCNPC xenograft compared to controls, and the quantification of relative staining intensity of IHC. Scale bar: 25 μm. Student’s t-test between tet-shRUNX1T1 and control in each marker. *p < 0.05, **p < 0.01, ***p < 0.001. **(F)** Immunoblot confirming RUNX1T1 overexpression in PRAD cell lines with FU-CGW as a control. **(G)** Cell viability assays in RUNX1T1 overexpressed PRAD compared to controls. ANOVA, 5 technical replicates, ****p < 0.0001. **(H)** Quantification of tumor weight of PRAD+RUNX1T1 tumors compared to control. Student’s t-test, *p < 0.05. **(I)** Representative images of IHC showing proliferation markers including Ki67 and phospho-Histone H3 were increased upon RUNX1T1 overexpression in PRAD, and quantifications of relative staining intensity. Scale bar: 25 μm.Student’s t-test between RUNX1T1 and CGW control in each cell line and each marker. *p < 0.05. **(J)** Cell cycle analysis in RUNX1T1 overexpressed PRAD compared to controls. ANOVA, 3 technical replicates, *p < 0.05, **p < 0.01, ***p < 0.001, ****p < 0.0001, ns: non-significant. **(K)** Representative image of colony formation assay with RUNX1T1 overexpressed PRAD compared to controls, and the quantification of colony formation efficiency. Student’s t-test between RUNX1T1 and CGW control in each cell line. *p < 0.05, ***p < 0.001.

A hallmark of SCNPC aggressiveness is rapid proliferation (Butler and Huang, 2021). To investigate the functional role of RUNX1T1 in SCNPC growth and survival, we suppressed RUNX1T1 expression using two independent approaches: CRISPR-Cas9-mediated knockout with single-guide RNAs targeting RUNX1T1 (sgRUNX1T1) and a doxycycline-inducible short-hairpin RNA system (tet-shRUNX1T1) (Supplementary Figure 2A; Figure 2B). Both strategies resulted in efficient RUNX1T1 suppression and significantly reduced SCNPC cell proliferation by up to 50% across multiple SCNPC cell lines (Supplementary Figure 2B), indicating that RUNX1T1 is required for SCNPC growth.

To further characterize the molecular consequences of RUNX1T1 depletion, we performed transcriptomic profiling following inducible RUNX1T1 knockdown using tet-shRUNX1T1 and control tet-shSCR cells. RUNX1T1 loss induced substantial transcriptional reprogramming in SCNPC cells, including altered expression of several tumor suppressor-associated genes such as MFAP4 and FGF21 (Dai et al., 2021; Mohammadi et al., 2022; Yu et al., 2025) (Supplementary Figure 3). Gene set enrichment analysis (GSEA) further demonstrated that RUNX1T1 depletion significantly affected hallmark and Gene Ontology (GO) pathways associated with cell proliferation and apoptosis in both NCI-H660 and MSKCC-EF1 cells (Figure 2A), supporting a critical role of RUNX1T1 in SCNPC survival and proliferation.

Consistent with these transcriptomic findings, RUNX1T1 knockdown induced apoptotic signaling, as evidenced by increased cleaved PARP in immunoblot (Figure 2B). Cell-cycle profiling additionally revealed a marked accumulation of cells in the sub-G0/G1 population following RUNX1T1 silencing (Figure 2C), further supporting apoptosis-associated growth inhibition. The functional requirement for RUNX1T1 was validated *in vivo* using tet-shRUNX1T1 SCNPC xenografts, in which RUNX1T1 knockdown significantly suppressed tumor growth (Figure 2D) and reduced tumor weight by approximately 50% within 18 days (Supplementary Figures 4A,B). Consistent with the *in vitro* observations, RUNX1T1-depleted xenografts displayed increased cleaved PARP and cleaved Caspase 3 expression together with reduced levels of proliferation markers, including phospho-Histone H3 (pHH3) and Ki67 (Figure 2E).

To complement our loss-of-function studies, we examined whether RUNX1T1 overexpression promotes tumor growth in PRAD cells. RUNX1T1 was ectopically expressed in PRAD cell lines (C4-2, CWR22, and LNCaP) using lentiviral transduction (Figure 2F). RUNX1T1 overexpression significantly enhanced PRAD cell proliferation *in vitro* (Figure 2G) and accelerated tumor growth *in vivo* (Figures 2H,I; Supplementary Figure 4C). Mechanistically, RUNX1T1 promoted cell-cycle by facilitating G1-to-S phase transition (Figure 2J) and enhanced clonogenic self-renewal capacity in colony formation assays (Figure 2K; Supplementary Figure 5A).

Collectively, these findings demonstrate that RUNX1T1 is essential for SCNPC cell survival and tumor maintenance, while also promoting proliferative capacity and tumor growth in PRAD cells.

### RUNX1T1 drives NED identity, small-cell histology, and stemness in SCNPC

**FIGURE 3:**
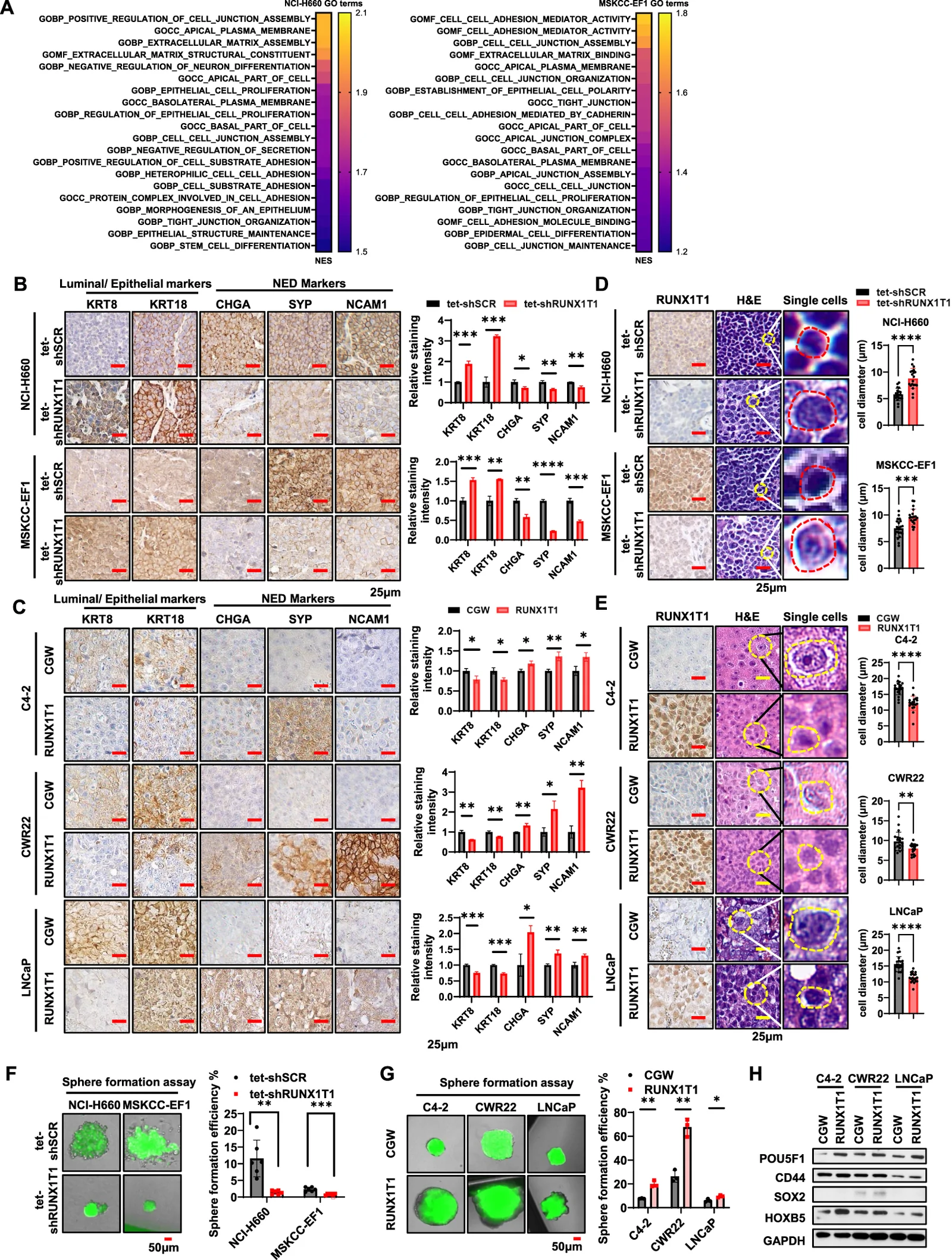
RUNX1T1 drives NE identity, small-cell histology, and stemness in SCNPC. **(A)** Selected GO terms enriched in GSEA analysis of SCNPC tet-shRUNX1T1 DEGs compared to controls, suggesting an enrichment of gain of epithelial identity and loss of NED identity. NES values were generated by GSEA software (Subramanian et al., 2005). **(B)** Representative IHC images of RUNX1T1 knockdown SCNPC compared to controls showing expression of epithelial markers (KRT8, KRT18) and NED markers (CHGA, SYP, and NCAM1), with quantification of relative staining intensity. Scale bar: 25 µm. Student’s t-test between RUNX1T1 knockdown and control in each marker, n = 3, *p < 0.05, **p < 0.01, ***p < 0.001, ****p < 0.0001. **(C)** Representative IHC images of RUNX1T1 overexpressed PRAD compared to controls showing expression of epithelial markers (KRT8, KRT18) and NED markers (CHGA, SYP, and NCAM1), with quantification of relative staining intensity. Scale bar: 25 µm. Student’s t-test between RUNX1T1 overexpressed and CGW control in each marker, n = 3, *p < 0.05, **p < 0.01, ***p < 0.001. **(D)** Representative IHC images of RUNX1T1 expression and H&E of RUNX1T1 knockdown SCNPC compared to controls, with quantification of cell diameter. Scale bar: 25 μm. Student’s t-test, n = 20, ***p < 0.001, ****p < 0.0001. **(E)** Representative IHC images of RUNX1T1 expression and H&E of RUNX1T1 overexpressed PRAD compared to controls, with quantification of cell diameter. Scale bar: 25 μm. Student’s t-test, n = 20, **p < 0.01, ****p < 0.0001. **(F)** Representative images of cell spheres in sphere formation assay with RUNX1T1 knockdown SCNPC compared to controls and the quantification of sphere formation efficiency. Scale bar: 50 μm. Student’s t-test between tet-shRUNX1T1 and control in each cell line, n = 6, **p < 0.01, ***p < 0.001. **(G)** Representative images of cell spheres in sphere formation assay with RUNX1T1 overexpressed PRAD compared to controls, and the quantification of sphere formation efficiency. Scale bar: 50 μm. Student’s t-test between RUNX1T1 and CGW control in each cell line, n = 3, *p < 0.05, **p < 0.01. **(H)** Immunoblot showing expression of stemness-associated markers (POU5F1, CD44, SOX2, HOXB5) in RUNX1T1-overexpressed PRAD compared to controls.

Compared with PRAD, SCNPC is characterized by neuroendocrine differentiation (NED) and loss of epithelial/luminal identity. Given our observation that RUNX1T1 is highly expressed in SCNPC relative to PRAD (Figures 1G,H), we next investigated whether RUNX1T1 contributes to lineage identity and NED. GSEA of our SCNPC RNA-seq following RUNX1T1 knockdown (Supplementary Figure 3) revealed significant alterations in biological processes associated with epithelial cell proliferation and morphogenesis, epidermal differentiation, apical-basal polarity, and cell-cell adhesion (Figure 3A). In parallel, pathways related to neuronal differentiation and stem cell differentiation were significantly affected (Figure 3A), suggesting that RUNX1T1 broadly regulates lineage plasticity and cell-state identity in SCNPC.

To validate these transcriptional changes, we examined the expression of well-established epithelial markers, KRT8 and KRT18 (Kirk et al., 2024; Wu et al., 2025). Both markers were significantly upregulated following RUNX1T1 depletion, indicating partial restoration of epithelial identity. In contrast, expression of canonical NED markers, including CHGA, SYP, and NCAM1, was markedly reduced upon RUNX1T1 knockdown especially *in vivo* (Figure 3B; Supplementary Figure 6). To further determine whether RUNX1T1 actively drives NED, we evaluated PRAD xenografts overexpressing RUNX1T1. Consistent with the loss-of-function studies, RUNX1T1 overexpression suppressed epithelial marker expression while inducing focal expression of NED markers (Figure 3C), supporting a role for RUNX1T1 in promoting neuroendocrine lineage reprogramming.

In addition to altering molecular lineage markers, RUNX1T1 manipulation also induced pronounced histological changes. RUNX1T1 knockdown in SCNPC xenografts resulted in loss of small-cell morphology, characterized by reduced cellular density, enlarged cell size, and decreased nuclear hyperchromasia (Figure 3D), indicating that RUNX1T1 is required to maintain the small-cell phenotype. Conversely, RUNX1T1 overexpression in PRAD xenografts promoted a more aggressive small-cell-like morphology, including reduced cell size and hyperchromatic nuclei (Figure 3E). Together with the increased proliferative activity observed in RUNX1T1-overexpressing tumors (Figure 2I), these findings suggest that RUNX1T1 promotes histologic transformation toward an aggressive SCNPC-like state.

Stemness-associated transcriptional programs are a well-recognized feature of SCNPC progression (Smith et al., 2015; Smith et al., 2018; Chen et al., 2023). Given the enrichment of stem cell differentiation pathways in RUNX1T1-regulated transcriptomes (Figure 3A), we next examined whether RUNX1T1 contributes to stem-like properties in prostate cancer. RUNX1T1 knockdown significantly impaired tumorsphere formation efficiency and growth in SCNPC models, whereas RUNX1T1 overexpression enhanced tumorsphere-forming in PRAD cells (Figures 3F,G). Consistent with these findings, RUNX1T1 depletion reduced expression of stemness-associated markers, including CD44, SOX2, and POU5F1, in SCNPC xenografts (Supplementary Figure 7A) (Palapattu et al., 2009; Zeineddine et al., 2014; Mamun et al., 2020). In contrast, RUNX1T1 overexpression increased expression of POU5F1 and the stemness-associated factor HOXB5 *in vitro* (Figure 3H), together with increased CD44 and POU5F1 expression *in vivo* (Supplementary Figure 7B) (Chen et al., 2016; Zhong et al., 2026).

Collectively, these findings establish RUNX1T1 as a key regulator of NED, small-cell histology, and stemness in SCNPC.

### RUNX1T1 is a key driver of SCNPC development in human prostate cancer

**FIGURE 4:**
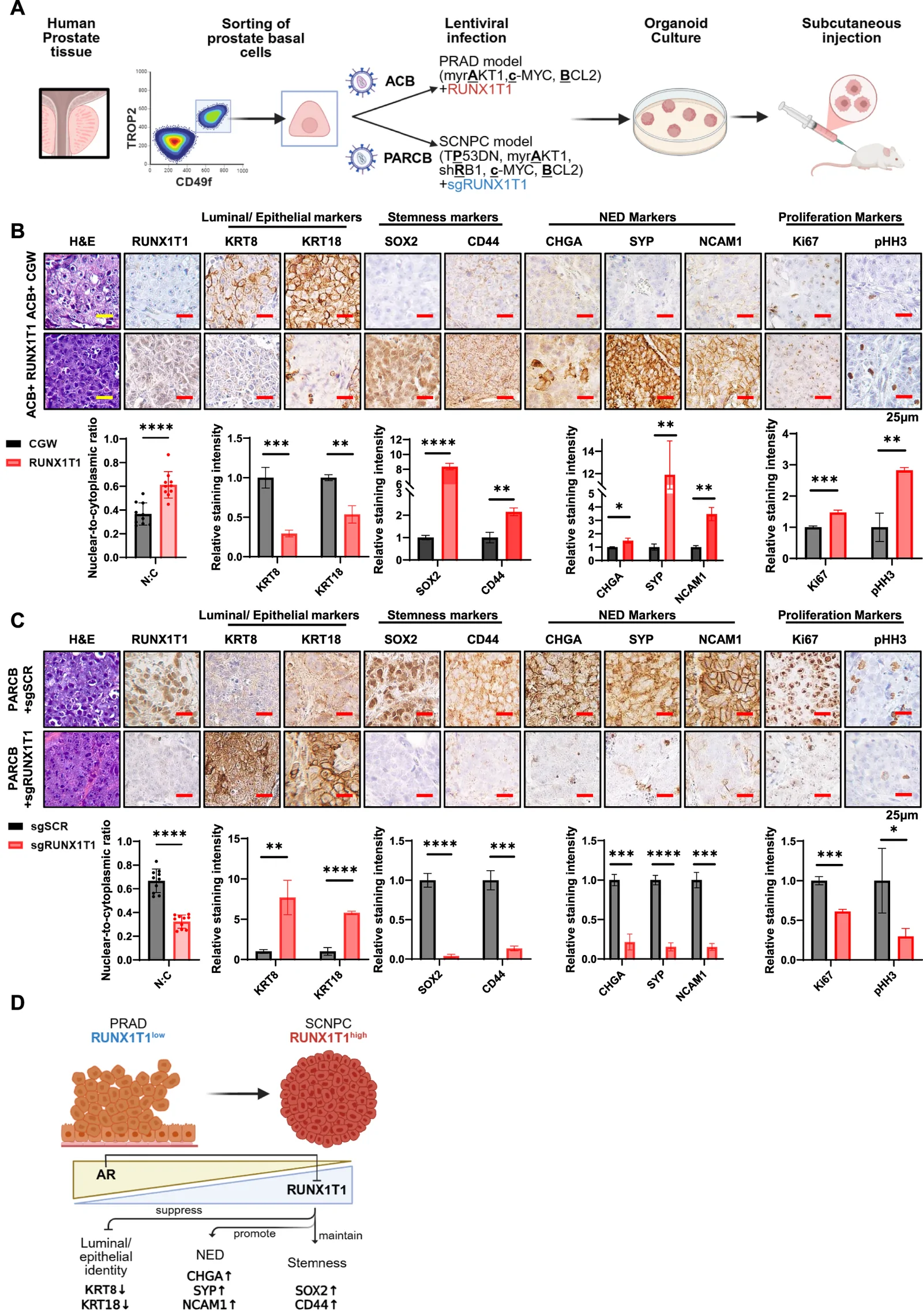
RUNX1T1 is a key driver of SCNPC development in human prostate cancer. **(A)** Schematic view of human cell reprogramming assay workflow. ACB models androgen-independent PRAD, while PARCB models stem-like SCNPC. **(B)** Representative H&E and IHC images of RUNX1T1 overexpressed ACB model compared to control ACB model showing histology, expression of RUNX1T1, epithelial markers (KRT8, KRT18), stemness markers (SOX2 and CD44), NED markers (CHGA, SYP, and NCAM1), and proliferation markers (Ki67 and pHH3). Quantifications of nuclear-to-cytoplasmic ratio and relative staining intensity were shown. Scale bar: 25 µm. Student’s t-test between RUNX1T1 overexpressed ACB tumor and CGW control ACB tumor in N:C ratio (n = 10) and each marker (n = 3), *p < 0.05, **p < 0.01, ***p < 0.001, ****p < 0.0001. **(C)** Representative H&E and IHC images of RUNX1T1 knockdown PARCB model compared to control PARCB model showing histology, expression of RUNX1T1, epithelial markers (KRT8, KRT18), stemness markers (SOX2 and CD44), NED markers (CHGA, SYP, and NCAM1), and proliferation markers (Ki67 and pHH3). Quantifications of nuclear-to-cytoplasmic ratio and relative staining intensity were shown. Scale bar: 25 µm. Student’s t-test between RUNX1T1 knockdown PARCB tumor and sgSCR control PARCB tumor in N:C ratio (n = 10) and each marker (n = 3), *p < 0.05, **p < 0.01, ***p < 0.001, ****p < 0.0001. **(D)** Schematic view of the key findings of this study.

Manipulation of RUNX1T1 expression in established PRAD and SCNPC cell lines altered lineage identity, as evidenced by changes in epithelial markers, NED markers, and histological features described above (Figures 2, 3). To further determine whether RUNX1T1 plays a critical role in the initiation of NED and histologic transformation during prostate cancer progression, we utilized a genetically defined human cell reprogramming model that recapitulates de novo progression from normal prostate cells to either PRAD or SCNPC *in vivo* (Figure 4A) (Park et al., 2018; Chen et al., 2023).

Using this system, we overexpressed RUNX1T1 in the ACB-PRAD model (Park et al., 2018) to determine whether RUNX1T1 is sufficient to initiate NED and small-cell transformation during tumor development. In parallel, we genetically deleted RUNX1T1 in the PARCB stem-like SCNPC model (Park et al., 2018) to assess whether RUNX1T1 is required for the establishment and maintenance of SCNPC identity during disease progression (Figure 4A).

Histopathological analysis demonstrated that RUNX1T1 overexpression in the PRAD model (ACB+RUNX1T1) was sufficient to induce a pronounced small-cell-like phenotype characterized by increased nuclear-to-cytoplasmic (N:C) ratio, hyperchromatic nuclei, scant cytoplasm, reduced cell size, and extensive nuclear molding (Figure 4B). These morphological changes were accompanied by marked suppression of luminal/epithelial markers KRT8 and KRT18 and induction of NED markers, including CHGA, SYP, and NCAM1 (Figure 4B). Consistent with our cell line studies, RUNX1T1 overexpression also markedly increased expression of stemness-associated markers SOX2 and CD44, together with proliferation markers Ki67 and pHH3 (Figure 4B), indicating that RUNX1T1 promotes the emergence of an aggressive stem-like SCNPC phenotype.

We next investigated whether RUNX1T1 is required for the acquisition of SCNPC features during tumor formation. Genetic deletion of RUNX1T1 at the onset of SCNPC development (PARCB+sgRUNX1T1) reversed the small-cell phenotype, resulting in reduced N:C ratio, decreased nuclear hyperchromasia, and restoration of epithelial marker expression, including KRT8 and KRT18 (Figure 4C). Importantly, RUNX1T1 loss abolished expression of both stemness-associated markers (SOX2 and CD44) and NED markers (CHGA, SYP, and NCAM1), thereby suppressing the stem-like SCNPC phenotype (Figure 4C). RUNX1T1 depletion additionally impaired tumor growth, as evidenced by reduced Ki67 and pHH3 expression (Figure 4C). Together, these findings demonstrate that RUNX1T1 is required for the establishment of aggressive stem-like SCNPC identity during prostate cancer progression.

Collectively, the pathological and molecular findings from this human prostate cancer reprogramming model identify RUNX1T1 as a key regulator of NED and histologic transformation into stem-like SCNPC during human prostate cancer development (Figure 4D).

## Discussion

Small-cell neuroendocrine prostate cancer (SCNPC) emerges in prostate cancer patients undergoing hormonal therapy and remains a major clinical challenge. Loss of androgen receptor (AR) signaling drives prostate adenocarcinoma (PRAD) toward a lineage-plastic and stem-like state (Smith et al., 2015; Malta et al., 2018; Smith et al., 2018), characterized by reduced AR dependency, acquisition of neuroendocrine differentiation (NED) features, and development of aggressive small-cell histology (Terry and Beltran, 2014). In the present study, we identify RUNX1T1 as a critical regulator of stem-like SCNPC identity. RUNX1T1 was highly upregulated in SCNPC, suppressed by AR, and functionally capable of bidirectionally regulating lineage identity in both PRAD and SCNPC models. These findings position RUNX1T1 at the intersection of stemness, epithelial, and NED plasticity during prostate cancer progression.

Our findings establish a mechanistic link between AR suppression and RUNX1T1 activation during lineage plasticity. AR signaling normally promotes luminal/epithelial differentiation while suppressing basal and stem cell-associated programs within the prostate epithelium (Kregel et al., 2013; Xie et al., 2017). Upon AR pathway inhibition, AR repression on RUNX1T1 expression was relieved, thereby permitting activation of transcriptional programs associated with stemness and NED, which is consistent with previous reports showing that AR suppression unleashes lineage-plastic programs regulated by factors such as EZH2, SOX2, and FOXA2 during SCNPC progression (Dardenne et al., 2016; Ku et al., 2017). However, manipulation of RUNX1T1 expression in PRAD and SCNPC does not change the expression of AR in turn, positioning AR upstream of RUNX1T1 (Supplementary Figure 8).

In addition to transcriptional derepression following AR loss, genomic amplification may further contribute to RUNX1T1 activation during advanced disease progression. RUNX1T1 is located on chromosome 8q (Canoy et al., 2023), the most frequently amplified autosomal chromosomal region in metastatic CRPC (Alshalalfa et al., 2023). The recurrent amplification suggests that genomic gain may cooperate with AR suppression to sustain elevated RUNX1T1 expression during SCNPC evolution.

Our functional studies indicate that RUNX1T1 is not only associated with SCNPC but is also required to establish and maintain its molecular and histological identity. RUNX1T1 depletion restored epithelial differentiation programs, including epithelial polarity and adhesion pathways, while suppressing neuroendocrine and stemness-associated features. These findings suggest that RUNX1T1 actively represses residual epithelial identity in SCNPC, thereby stabilizing the lineage-plastic state and preventing reversion toward a differentiated PRAD phenotype. They also highlight the metastable nature of SCNPC identity, consistent with prior models of lineage plasticity in which tumor cell states remain reversible upon disruption of key regulatory factors (Quintanal-Villalonga et al., 2020).

Importantly, complementary gain- and loss-of-function studies demonstrated that RUNX1T1 bidirectionally controls SCNPC-associated phenotypes, including NED, small-cell morphology, proliferation, and stemness. RUNX1T1 overexpression sufficiently induced small-cell-like histology and stemness programs in PRAD models, whereas RUNX1T1 depletion reversed these features in SCNPC. Its functional behavior resembles that of established SCNPC lineage regulators such as ASCL1, MYCN, and EZH2 (Beltran et al., 2016; Cyrta et al., 2020), supporting that RUNX1T1 functions as a central regulator of SCNPC lineage identity.

Mechanistically, ChIP-qPCR analysis demonstrated that RUNX1T1 binds the promoters of the epithelial markers KRT8 and KRT18 in SCNPC, exhibiting greater than two-fold enrichment relative to IgG controls (Supplementary Figure 9A). These findings are consistent with the established role of RUNX1T1 as a transcriptional co-repressor in acute myeloid leukemia and neuroblastoma (Melnick et al., 2000; Murray et al., 2024). In contrast, RUNX1T1 enrichment was not consistently detected at the promoters of neuroendocrine differentiation (NED) or stemness-associated genes (Supplementary Figures 9B,C). Specifically, modest enrichment at the CHGA and SYP promoters was observed in MSKCC-EF1 cells but not in NCI-H660 cells (Supplementary Figure 9B). This cell line-dependent promoter occupancy may reflect the molecular heterogeneity of SCNPC consistent with the variable expression of NED markers reported in clinical SCNPC specimens (Yao et al., 2006; Lee et al., 2016). Collectively, these findings suggest that RUNX1T1 may directly repress epithelial lineage genes while regulating neuroendocrine programs through indirect or context-dependent mechanisms. Future studies combining unbiased identification of RUNX1T1-interacting proteins with genome-wide mapping of RUNX1T1 chromatin occupancy (e.g., ChIP-seq or CUT&RUN) will be important for defining the transcriptional networks controlled by RUNX1T1 during SCNPC development.

Overall, this study provides new insight into the molecular events initiated by AR pathway inhibition that drive SCNPC progression. Using our *in vivo* human prostate cell reprogramming model, we demonstrate that RUNX1T1 is sufficient to promote both neuroendocrine and small-cell transformation and necessary for maintenance of stem-like SCNPC identity. These findings may also have important translational implications. First, because RUNX1T1 expression strongly correlates with SCNPC progression, it may serve as a biomarker for identifying lineage-plastic PRAD subclones at risk for neuroendocrine/small-cell transformation. Second, because RUNX1T1 activation occurs downstream of AR inhibition and contributes to NED and stemness, therapeutic targeting of RUNX1T1 or its downstream transcriptional programs may represent a potential strategy to delay or prevent SCNPC progression during castration resistance.

## Methods

### Lentiviral sgRNAs and shRNAs

Plasmids with shRNA targeting RUNX1T1 (shRUNX1T1: TRCN0000271236, 5′- CAA​GCG​ACC​ATG​CAC​TAT​TAG-3′) and Scramble (shSCR: 5′-CCT​AAG​GTT​AAG​TCG​CCC​TCG-3′) were based on Tet-pLKO-CMV-GFP vector, designed by adding CMV-GFP downstream puro-resistance gene in Tet-pLKO-puro (Addgene, Cat.#21916). Plasmids to knockout RUNX1T1 (#1: TGG​CCA​GTC​TTG​CGC​AGT​GG, #2: TCC​AAA​CGG​TAA​TGC​TGA​GG), AR (CAG​CCT​AAC​CAG​GCG​GGT​CG), and Scramble were based on TLCV2 (Addgene, Cat.#87360). RUNX1T1 cDNA from NCI-H660 was cloned into FUCGW plasmid from Dr. Owen Witte at the University of California, Los Angeles. Plasmids were co-transfected with pMDL, pREV, and pVSV-G into HEK-293T cells (ATCC, Cat.#CRL-3216), in DMEM (10%FBS, 1%penicillin/streptomycin (P/S)) for lentivirus. Lentiviruses were delivered to cells as described (Park et al., 2018).

### RNA-seq library preparation, sequencing, and analysis

RNA was isolated from MSKCC-EF1 and NCI-H660 tet-shRUNX1T1 using the QIAzol (Cat.#79306) and RNeasy Mini Kit (Qiagen, Cat.#74104). Library was prepared with NEBNext Ultra II Kit (Cat.#E3330S). RNA integrity was verified by TapeStation (Agilent Technologies, Palo Alto, CA, United States). Sequencing was performed at Admera Health, LLC. (South Plainfield, NJ, United States). Reads were trimmed with Trimgalore (https://github.com/FelixKrueger/TrimGalore) and aligned to the reference genome (hg38) using STAR (Dobin et al., 2013). Gene reads were quantified using HTseq (Anders et al., 2015). Differentially expressed genes (DEGs) were identified using DESeq2 (Love et al., 2014) (adjusted p-value < 0.05). GSEA analyses were performed with DEGs as described (Park et al., 2016; Park et al., 2018).

### Cell culture and proliferation assays

PRAD cell lines (C4-2, CWR22, LNCaP, DU145, and PC3) were cultured in RPMI-1640 (10%FBS and 1%P/S) with full serum. MSKCC-EF1 and NCI-H660 cell lines were categorized as SCNPC by prior studies (Johnson et al., 1989; Smith et al., 2018; Corella et al., 2020) and histologically confirmed by our pathologist, Dr. Jiaoti Huang. They were cultured in serum-free stem cell media (Advanced-DMEM/F-12, B27, 1%P/S, 10 ng/mL hEGF, 10 ng/mL FGF2, 10 μg/mL Normocin and 5 mL GlutaMAX(100X)). For cell proliferation assay: 5 × 10^4^ cells of C4-2, CWR22, and LNCaP transduced with RUNX1T1 or CGW were plated in flat-bottom 96-well plates, cultured for 5 days at 37 °C, 5%CO_2_ in 100 µL of RPMI-1640 media. For SCNPC, 5 × 10^4^ SCNPC cells were plated, transduced with sgSCR or sgRUNX1T1, and cultured for 5 days at 37 °C, 5%CO_2_ in 100 µL of stem cell media. Cell proliferation was measured with CellTiter-Glo® 2.0 (Promega, Cat.#G7572).

### Sphere and colony formation assays

In sphere formation assay (Bahmad et al., 2018), 400 cells were mixed with 50 μL Matrigel (Corning, Cat.#356234) and spread along the rim of clear flat-bottom-24-well plates, cultured for 30 days in 200 μL stem cell media. For SCNPC tet-shRUNX1T1, doxycycline was provided at 2 μg/mL every 2 days. In colony formation assay, 400 cells were plated in a 6-well plate with 2 mL of stem cell media. After 14 days, colonies were fixed with 100% Methanol and stained with 0.2% crystal violet in PBS.

### Cell cycle analysis by propidium iodide (PI) staining

1 × 10^6^ cells were fixed with 70% ice-cold ethanol, washed with ice-cold PBS, and resuspended in PBS containing RNase (10 μg/mL), PI (50 μg/mL), and 0.5% Triton X-100. Cell cycles were measured by BD FACSCanto™ II and analyzed in FlowJo.

### Cell lysate collection and immunoblot assay

Cells were lysed in 9M urea buffer with protein amount measured (Prometheus, Cat.#18441). Samples were subjected to SDS-PAGE and transferred to nitrocellulose membranes (Prometheus, Cat.#84-875), Used primary antibodies are RUNX1T1: Proteintech (Cat.#15494-1-AP), Androgen Receptor: Cell signaling Technology (Cat.#D6F1T), GAPDH: GeneTex (Cat.#GTX627408), POU5F1/OCT4: MyBioSource (Cat.#MBS1491031), CD44: Abcam (Cat.#ab157107), SOX2: Proteintech (Cat.#11064-1-AP), HOXB5: Abcam (Cat.#ab109375), Cleaved PARP: Cell Signaling Technology (Cat.#94885S), Beta-tubulin: Proteintech (Cat.#66240-1-Ig), Alpha-tubulin: Cell Signaling Technology (Cat.#2144S), Beta-actin: Cell Signaling Technology (Cat.#4970S), NCAM1: Cell Signaling Technology (Cat.#99746S), CHGA: Cell Signaling Technology (Cat.#85798S), SYP: Cell Signaling Technology (Cat.#36406S). Membranes were incubated with secondary antibodies for 2 h at room temperature. ECL reagent was applied (Prometheus, Cat.#20–300B) and bands were visualized using X-ray films (Prometheus, Cat.#30–810L).

### Xenograft tumor development

4 × 10^6^ cells (C4-2, CWR22, and LNCaP: RUNX1T1 vs. CGW; MSKCC-EF1 and NCI-H660: tet-shRUNX1T1 vs. tet-shSCR) were subcutaneously injected with 50 μL Matrigel (Corning, Cat.#356234) into 8–12-week-old male immunodeficient mice (NSG). For PRAD, tumors were harvested after 4 weeks. For SCNPC, mice were treated with a doxycycline diet when tumors reached 200 mm^3^. Tumor size was measured every 2 days. Tumors were harvested before reaching 2000 mm^3^.

### Tissue processing and immunohistochemistry

Tumors were preserved in 10% buffered formalin, then processed (Expedia™ STP 120 Spin Tissue Processor), cut into 5 μm, and deparaffinized. Antigen retrieval was done by heating in 10 mM sodium citrate buffer (pH 6.0) for 30 min. We used primary antibodies described above and Ki67: Abcam (Cat.# ab15580), pHH3: Cell Signaling Technology (Cat.#53348S), KRT8: BioLegend (Cat.#MMS-162P), KRT18: GenuIN (Cat.#2214), POU5F1: ABclonal (Cat.#A7920). Sections were incubated with ImmPRESS HRP Anti-Rabbit or Mouse IgG reagent (Vector, Cat.#LS-J1066-15, Cat.#MP-7402). Immunohistochemistry was performed with the ImmPACT DAB Peroxidase (HRP) Substrate kit (Vector, Cat.#SK-4105) and counterstained with hematoxylin, and mounted (Thermo Fisher, Cat.#22-050-262).

### Chromatin immunoprecipitation (ChIP), dual cross-linking ChIP, and qPCR

ChIP assays to check AR enrichment at RUNX1T1 promoter was performed as in Chen et al. (2021). For dual cross-linking ChIP to check RUNX1T1 enrichment at promoters of luminal/epithelial markers, NED markers, and stemness markers, 2 mM EGS (ethylene glycol bis (succinimidyl succinate)) (Thermo Scientific Cat.#21565) treatment for 20 min was followed by regular ChIP. qPCR with SYBR Green qPCR Master Mix Low ROX (MedChemExpress Cat.#HY-K0522) was done. Primers are listed in Supplementary Table 1.

### Human prostate cell reprogramming assay

Protocols for processing human prostate tissue were as described (Park et al., 2018). Human prostate tissue was provided by the Duke BioRepository and Precision Pathology Center.

### Animal protocol

NSG mice were from Jackson Laboratories and bred under the Duke Animal Care and Use program. Male mice were used. Injections of cells were performed according to protocols approved by Duke’s Animal Research Committee.

### Statistics

Statistical analyses were done in GraphPad Prism version 10. For Student’s t-test and ANOVA, a p-value less than 0.05 is statistically significant.

## Data Availability

The datasets presented in this study can be found in Gene Expression Omnibus (GEO) with accession number GSE330171.
